# Efficacy and safety of combined immunotherapy and antiangiogenic therapy for advanced non-small cell lung cancer: a real-world observation study

**DOI:** 10.1186/s12890-023-02470-z

**Published:** 2023-05-19

**Authors:** Ke Ma, Qianqian Guo, Xingya Li

**Affiliations:** Department of Medical Oncology, The First Affiliated Hospital of Zhengzhou University, No.50 Building East Road, Zhengzhou, Henan 450052 People’s Republic of China

**Keywords:** Immunotherapy, Antiangiogenic therapy, Chemotherapy-free, Non-small cell lung cancer

## Abstract

**Purpose:**

This study was performed to investigate the efficacy and safety of combined immunotherapy and antiangiogenic therapy for advanced non-small cell lung cancer (NSCLC) in the real world.

**Methods:**

Data on clinicopathological features, efficacy and adverse events (AEs) were collected retrospectively in advanced NSCLC patients who received immunotherapy combined with antiangiogenic therapy.

**Results:**

A total of 85 advanced NSCLC patients were enrolled. The patients had a median progression-free survival (PFS) of 7.9 months and a median overall survival (OS) of 18.60 months. The objective response rate and disease control rate were 32.9% and 83.5%, respectively. Subgroup analysis revealed that NSCLC patients with stage IV (*p* = 0.042), brain metastasis (*p* = 0.016) and bone metastasis (*p* = 0.016) had shorter PFS. NSCLC patients with brain metastasis (*p* = 0.025), liver metastasis (*p* = 0.012), bone metastasis (*p* = 0.014) and EGFR mutations (*p* = 0.033) had shorter OS. Multivariate analysis revealed that brain metastasis (HR = 1.798, 95% CI: 1.038, 3.112, *p* = 0.036) and bone metastasis (HR = 1.824, 95% CI: 1.077, 3.090, *p* = 0.025) were independent predictive factors of PFS, and bone metastasis (HR = 2.00, 95% CI: 1.124, 3.558, *p* = 0.018) was an independent predictive factor of OS. In addition, patients receiving immunotherapy combined with antiangiogenic therapy in second-line therapy had longer OS than those receiving immunotherapy in third- or later-line therapy (*p* = 0.039). Patients with EGFR mutations who received combination therapy had worse OS than those with KRAS mutations (*p* = 0.026). Furthermore, PD-L1 expression was associated with treatment responses in advanced NSCLC (χ2 = 22.123, *p* = 0.000). AEs of different grades occurred in 92.9% (79/85) of NSCLC patients, most of which were mild grade 1/2 AEs. No grade 5 fatal AEs occurred.

**Conclusion:**

Immunotherapy combined with antiangiogenic therapy was an option for advanced NSCLC patients with good safety and tolerability. Brain metastases and bone metastases were potentially independent negative predictors of PFS. Bone metastases were a potential independent negative predictor of OS. PD-L1 expression was a potential predictor of response for immunotherapy combined with antiangiogenic therapy.

## Introduction

Lung cancer is still the most common malignant tumour with morbidity and mortality in China and seriously threatens the life and health of Chinese people [1]. Non-small cell lung cancer (NSCLC) accounts for approximately 85% of lung cancers. In recent years, with the continuous progress of molecular biology technology, NSCLC has been increasingly identified as a highly heterogeneous disease. Targeted therapy and immunotherapy for different molecular types have greatly improved the prognosis of patients [2]. Especially for advanced NSCLC patients without targetable driver oncogenes, immune checkpoint inhibitors (ICIs) provide new therapeutic options with longer progression-free survival (PFS) and overall survival (OS) [3]. However, the overall effective rate of ICI monotherapy in NSCLC is only 20% [4]. How to obtain the dominant population of immunotherapy and improve the efficacy of immunotherapy is a hot topic in clinical research.

Antiangiogenic drugs, including monoclonal antibodies targeting vascular endothelial growth factor (VEGF) or VEGF receptors (VEGFRs), such as bevacizumab [5, 6], and small-molecule tyrosine kinase inhibitors (TKIs) targeting multiple angiogenic pathways, such as anlotinib [7, 8] and apatinib [9, 10], have shown antitumour effects in NSCLC.

A preclinical study revealed that tumour angiogenesis is closely related to the immune microenvironment [11]. Tumour vascular normalization and immune reprogramming form a reinforcing loop that reconditions the tumour immune microenvironment to induce durable antitumour immunity [12]. Antiangiogenic therapy can normalize the blood vessels in this part of the tumour, weaken the suppression of immune factors, and thus promote the development of immunotherapy, which is beneficial for the application of immunotherapy. Additionally, ICIs can normalize the tumour vascular system by activating effector T cells and increasing the infiltrating and killing functions of effector T cells [12].

At present, a large number of clinical studies have explored the efficacy of the model of ICIs combined with antiangiogenic therapy in a variety of tumours and have observed good results [11, 13]. Impower150 is the first successful Phase III clinical study of the efficacy of immunotherapy combined with antiangiogenic therapy in NSCLC [14]. The results show that the addition of immunization to antiangiogenic therapy can significantly improve patients' OS (19.5 vs. 14.7 months; hazard rate [HR] 0.80; 95% confidence interval [CI] 0.67–0.95) [15]. Based on this, antiangiogenic therapy combined with immunotherapy (programmed death ligand 1 [PD-L1] inhibitor) and chemotherapy has been approved by the FDA as first-line treatment for advanced NSCLC patients. The phase III ORIENT-31 study also proved the clinical efficacy of chemotherapy combined with immunotherapy (programmed death-1[PD-1] inhibitor) and antiangiogenic therapy [16].

However, a higher number of drug combinations is associated with a greater economic burden and a relatively higher incidence of adverse events (AEs) despite improving treatment efficacy [15, 16]. Especially for elderly patients with poor general status, aggressive chemotherapy is often intolerable [17]. In 2022, ESMO published the results of the Phase III IPSOS study of first-line atezolizumab vs. single-agent chemotherapy in patients with NSCLC who were not eligible for platinum-containing chemotherapy, showing that compared with chemotherapy, first-line ICI had an OS benefit (HR = 0.78; 95% CI: 0.6, 0.97; *p* = 0.028) with stable health-related quality of life and good tolerance [18]. Chemotherapy-free models are increasingly popular. At present, a number of clinical studies on chemotherapy-free models have been carried out in NSCLC patients, showing promising clinical significance [19]. Therefore, we retrospectively analysed 85 patients with advanced NSCLC who received ICIs combined with antiangiogenic drugs and evaluated the efficacy and safety of this chemotherapy-free combination regimen in the real world to provide more options and a basis for the treatment of advanced NSCLC patients.

## Methods

### Patients

Patients were enrolled according to the following inclusion criteria: (1) patients were pathologically diagnosed with advanced or metastatic NSCLC in the First Affiliated Hospital of Zhengzhou University; (2) ICIs combined with antiangiogenic therapy were used during the treatment and regardless of the treatment lines from March 1, 2019, to September 30, 2021; (3) there were measurable lesions according to Response Evaluation Criteria of Solid Tumours (RECIST) 1.1 version [20]; and (4) all patients agreed to participate in the study and signed informed consent.

Patients were excluded for the following reasons: (1) patients with other malignant tumours that were not cured in five years; (2) chemotherapy was combined with ICIs and antiangiogenic therapy.

### Treatment

The ICIs included pembrolizumab, camrelizumab, sintilimab, tislelizumab and toripalimab. Patients were treated with pembrolizumab, camrelizumab, sintilimab or tislelizumab at a dose of 200 mg every three weeks. Toripalimab was administered at a dose of 240 mg every 3 weeks. The antiangiogenic drugs included bevacizumab, anlotinib, and apatinib. Bevacizumab was administered at a dose of 15 mg/kg every three weeks. Anlotinib was administered at a dose of 12 mg/10 mg/8 mg depending on the tolerance of patients for two weeks and stopped for one week. Apatinib was administered at a dose of 250 mg daily.

### Efficacy and safety

The assessment of treatment efficacy was based on RECIST version 1.1 [20]. The tumour responses of target lesions were divided into complete response (CR), partial response (PR), stable disease (SD) and progressive disease (PD). Objective Response Rate (ORR) = CR + PR/total number of enrolled cases; Disease Control Rate (DCR) = CR + PR + SD/total enrolled cases. PFS was defined as the time from the start of combination therapy to PD or death from any cause. OS was defined as the period from the start of combination therapy until death from any cause or the last follow-up. The deadline for follow-up was August 31, 2022. AEs were evaluated and recorded according to the Common Terminology Criteria Adverse Events (CTCAE) V5.0.

### Statistical analysis

Survival curves and median PFS and OS were generated using the Kaplan‒Meier survival method. Risk factors for subgroups were calculated using the Cox proportional hazards regression model.

Multivariate analyses were based on the Cox proportional hazards regression model. Clinical treatment responses were analysed using χ2 tests. All statistical analyses were performed using SPSS 26.0 statistical software, and *p* < 0.05 was considered statistically significant.

## Results

### Patient characteristics

A total of 85 NSCLC patients who received a combination of ICIs and antiangiogenic drugs were enrolled in the study (Table 1). The median age was 62 (ranging from 33 to 90). The male-to-female ratio was 3.5:1 (66 cases and 19 cases, respectively). PD-L1 expression was evaluated in all patients by immunohistochemistry with the 22C3 assay. All of the 48 lung adenocarcinoma patients underwent genetic testing: 7 patients (14.6%) harboured epidermal growth factor receptor (EGFR) exon 21 L858R mutations, 4 patients (8.3%) harboured EGFR exon 19 deletion mutation, 2 patient (4.2%) harboured primary EGFR exon 20 T790 mutation, 3 patients (6.3%) harboured EGFR nonclassical mutations (exon 21 L861Q and exon 18 deletion mutation, exon 20 insertion mutation), 2 patients (4.2%) harboured anaplastic lymphoma kinase (ALK) fusion, 2 patients (4.2%) harboured ROS proto-oncogene 1 (ROS1) fusion, 2 patient (4.2%) harboured v-raf murine sarcoma viral oncogene homologue B (BRAF) V600E mutation, 13 patients (27.1%) harboured kirsten rat sarcoma viral oncogene homologue (KRAS) mutation and another 2 patients (4.2%) harboured epidermal growth factor receptor 2 (HER-2) exon 20 insertion mutation. Among the 37 squamous cell carcinoma NSCLC patients, 19 patients underwent genetic testing and 1 patient (2.7%) harboured EGFR exon 19 deletion mutation and 1 (2.7%) patient harboured KRAS-L19F mutation.

**Table 1:** Baseline characteristics of 85 NSCLC patients

Variables	No	%
Gender		
Male	66	77.65
Female	19	22.35
Ages		
< 60 years	40	47.06
≥60 years	45	52.94
Histology		
Adenocarcinoma	48	56.47
Squamous cell carcinoma	37	43.53
AJCC TNM Stage		
Stage III	13	15.29
Stage IV	72	84.71
Brain metastasis		
Yes	22	38.82
No	63	61.18
Liver metastasis		
Yes	15	17.65
No	70	82.35
Bone metastasis		
Yes	33	38.82
No	52	61.18
Pleural effusion		
Yes	14	16.47
No	71	83.53
Treatment line		
1 line	5	5.88
2 lines	35	41.18
≥ 3 lines	45	52.94
PD-L1 expression		
Negative	44	51.76
Low	28	32.94
High	13	15.29
Actionable Driver Mutation		
Classical EGFR	14	16.47
Unclassical EGFR	3	3.53
ALK Fusion	2	2.35
ROS1	2	2.35
BRAF-V600E	2	2.35
HER-2 20 insertion	2	2.35
KRAS	14	16.47
KRAS-G12C	5	5.88
RAS-G12A	3	3.53
KRAS-G12V	3	3.53
KRAS-G12D	1	1.18
KRAS-D119	1	1.18
KRAS-L19F	1	1.18
History of smoke		
Yes	41	48.24
No	44	51.76

*NSCLC* Non-small cell lung cancer, *EGFR* Epidermal growth factor receptor, *ALK* Anaplastic lymphoma kinase, *ROS1* ROS proto-oncogene 1, *B-RAF* v-raf murine sarcoma viral oncogene homologue B, *HER-2* Epidermal growth factor receptor 2, *KRAS* Kirsten rat sarcoma viral oncogene homologue. PD-L1 (programmed death ligand 1) expression, Negative, 0%; Low, 1%-49%; High, ≥ 50%

### Treatment strategies

In total, 5 patients (5.88%) received combination therapy with PD-1 inhibitors and antiangiogenic agents as first-line treatment, 35 patients (41.18%) received the combination regimen as second-line treatment, and 45 patients (52.94%) received the combination regimen as third-line or later treatment. The most frequently used ICIs and angiogenetic drugs were camrelizumab (71.76%) and anlotinib (68.23), respectively (Table 2).

**Table 2:** Drugs used in the combination treatment of PD-1 inhibitors and antiangiogenic treatment

Agents of combination strategy	n (%)
Camrelizumab + anlotinib	46 (54.11)
Camrelizumab + apatinib	15 (17.65)
Camrelizumab + bevacizumab	5 (5.88)
Sintilimab + anlotinib	5 (5.88)
Sintilimab + apatinib	4 (4.71)
Pembrolizumab + anlotinib	4 (4.71)
Pembrolizumab + apatinib	2 (2.35)
Toripalimab + anlotinib	3 (3.53)
Tislelizumab + anlotinib	1 (1.18)

*PD-1* Programmed death 1

### PFS and OS

**Fig. 1:**
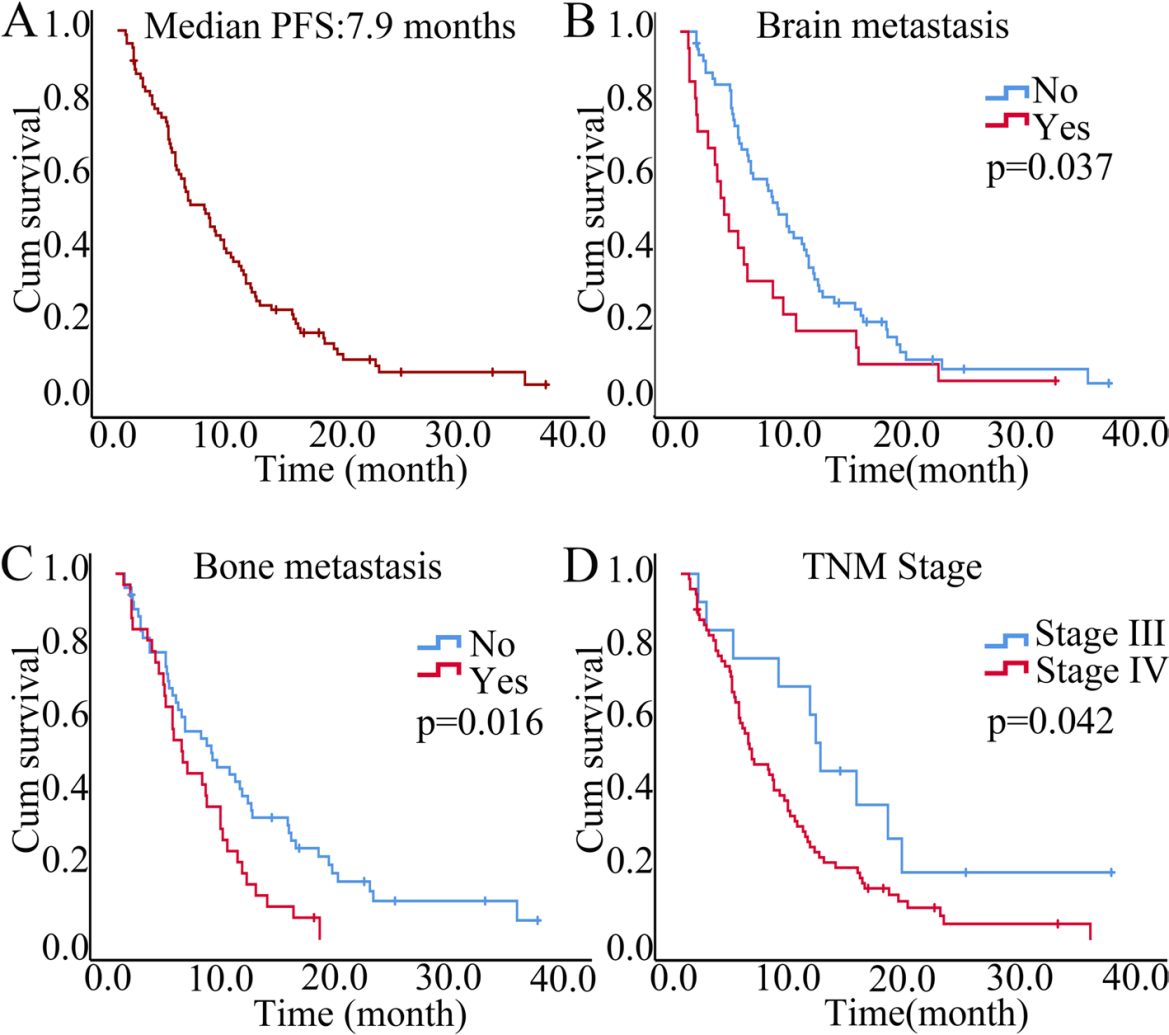
Kaplan–Meier curves for progression-free survival (PFS) (**A**) and stratified by clinical characteristics, including (**B**) brain metastasis, (**C**) bone metastasis, and (**D**) TNM stage

**Fig. 2:**
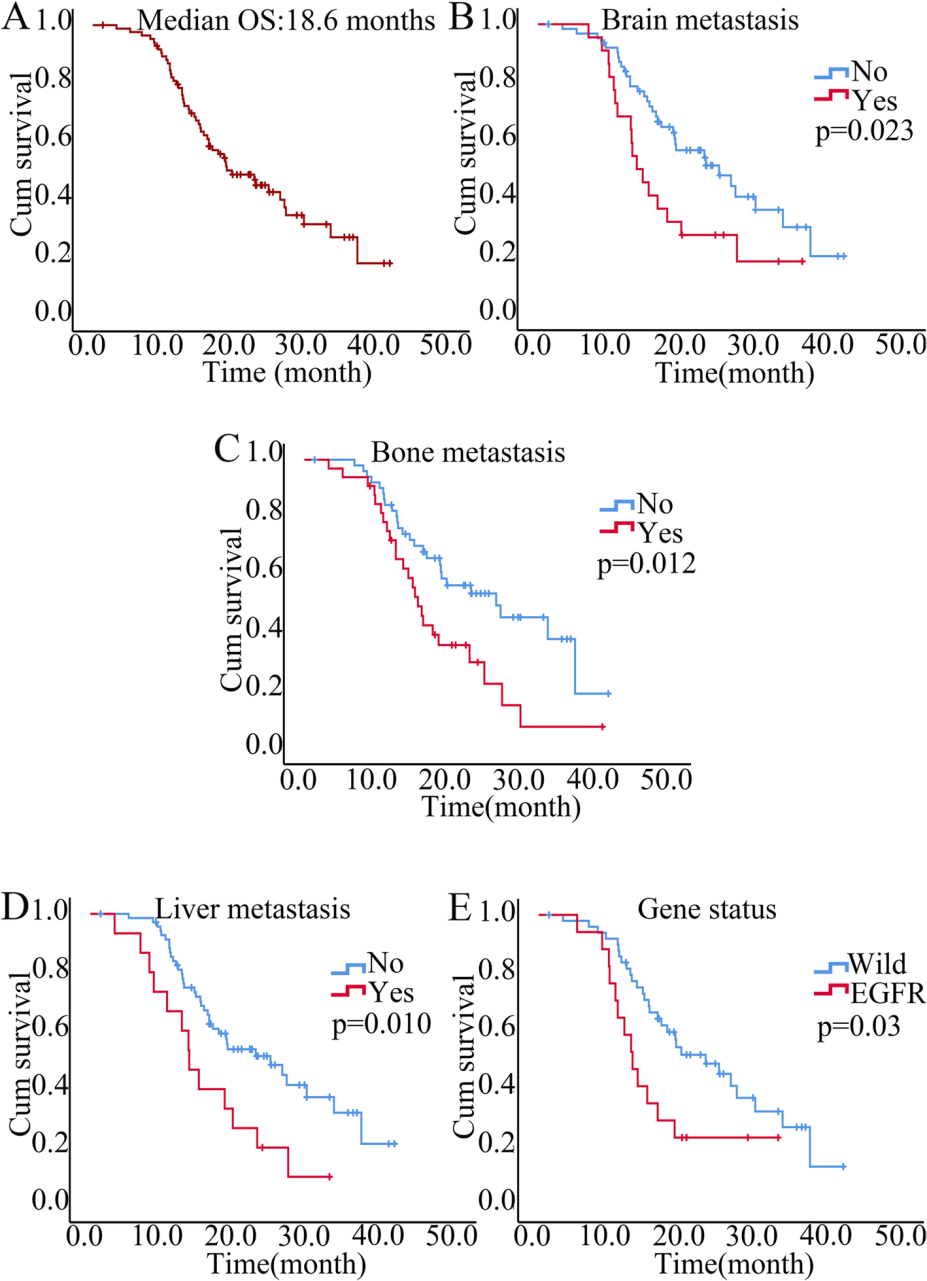
Kaplan–Meier curves for overall survival (OS) (**A**) and stratified by clinical characteristics, including (**B**) brain metastasis, (**C**) bone metastasis, (**D**) liver metastasis, (**E**) gene status. Note: EGFR (epidermal growth factor receptor), including classical EGFR mutation and unclassical EGFR mutation

By the time of data cut-off, the median follow-up was 17.4 months. The median PFS (mPFS) of 85 NSCLC patients was 7.9 months (95% CI: 5.568, 10.232), and the median OS (mOS) was 18.60 months (95% CI: 13.24, 23.96) (Figs. 1A and 2A). Subgroup analysis showed that NSCLC patients with stage IV (*p* = 0.042), brain metastasis *(p* = 0.037) and bone metastasis (*p* = 0.016) who received combination therapy with PD-1 inhibitors and antiangiogenic drugs had shorter PFS (Table 3 and Fig. 2B-D).

**Table 3:** Subgroup analysis of mPFS of advanced NSCLC patients who received combination therapy of PD-1 inhibitors and antiangiogenic drugs

Characters	mPFS(months)	HR (95% CI)	*p* value
Gender			
Male vs. Female	8.5 vs. 4.2	0.738(0.425,1.282)	0.281
Age			
< 60 vs. ≥ 60	7.9 vs. 7.6	0.931(0.592,1.464)	0.757
TNM Stage			
IV vs. III	6.1 vs. 12.0	2.005(1.026,3.920)	0.042*
Histology			
Adenocarcinoma vs. Squamous	5.9 vs. 9.8	1.568(0.994,2.474)	0.053
Smoke			
Yes vs. No	8.0 vs. 7.6	0.865(0.551,1.358)	0.528
Brain metastasis			
Yes vs. No	3.8 vs. 9.2	1.714(1.034,2.843)	0.037*
Liver metastasis			
Yes vs. No	5.0 vs. 8.4	1.320(0.738,2.362)	0.350
Bone metastasis			
Yes vs. No	5.9 vs. 8.5	1.802(1.118,2.902)	0.016*
Pleural effusion			
Yes vs. No	4.6 vs. 8.4	1.456(0.782,2.708)	0.236
Treatment lines			
1 line vs. 2 lines vs. ≥ 3 lines^a^	8.0 vs. 9.4 vs. 5.5	0.755(0.297, 1.915)	0.554
		0.659(0.410, 1.060)	0.086
PD L1 expression			
Negative vs. Positive	5.8 vs. 9.2	1.041(0.664,1.633)	0.859
Negative vs. Low. vs. High^a^	5.8 vs. 8.0 vs. 10.7	1.080 (0.561,2.079)	0.818
		1.192 (0.597,2.381)	0.619
Gene mutation			
Wild vs. all EGFR	8.0 vs. 4.4	0.567(0.318,1.010)	0.054
Wild vs. all EGFR vs. KRAS^a^	6.1 vs. 4.4 vs. 9.4	0.914(0.483,1.729)	0.783
		1.656(0.800,3.432)	0.174

*NSCLC* Non-small cell lung cancer, *mPFS* Median progression-free survival, *PD-1* Programmed death 1, *HR* Hazard rate, *CI* Confidence interval, *Squamous* Squamous cell carcinoma, *PD-L1* (programmed death ligand 1) expression, negative, 0%; positive, ≥ 1%, low, 1%-49%; high, ≥ 50%. *EGFR* (epidermal growth factor receptor), including classical EGFR mutation and nonclassical EGFR mutation, *KRAS* Kirsten Rat Sarcoma Viral Oncogene Homologue

^*^significance *p* values

^a^The last group served as a control

Patients with brain metastasis (*p* = 0.025), liver metastasis (*p* = 0.012), bone metastasis (*p* = 0.014) and EGFR mutations (*p* = 0.033) who received combination therapy with PD-1 inhibitors and antiangiogenic drugs had shorter OS (Table 4 and Fig. 2B-E). Furthermore, by introducing uni-variants with *p* < 0.05, multivariate Cox regression showed that brain metastasis (HR = 1.798, 95% CI: 1.038, 3.112, *p* = 0.036) and bone metastasis (HR = 1.824, 95% CI: 1.077, 3.090, *p* = 0.025) were independent predictive factors of PFS, and bone metastasis (HR = 2.00, 95% CI: 1.124, 3.558, *p* = 0.018) was an independent predictive factor of OS. Additionally, we found that patients receiving immunotherapy combined with antiangiogenic therapy in second-line therapy had longer OS than those in third- or later-line therapy (*p* = 0.039) (Table 4). Patients with EGFR mutations who received combination therapy had worse OS than those with KRAS mutations (*p* = 0.026) (Table 4).

**Table 4:** Subgroup analysis of the mOS of advanced NSCLC patients who received combination therapy with PD-1 inhibitors and antiangiogenic agents

Characters	mOS (months)	HR (95% CI)	*p* value
Gender			
Male vs. Female	24.4 vs. 13.4	0.563(0.302,1.048)	0.070
Age			
< 60 vs. > 60	15.9 vs. 22.6	1.354(0.775,2.364)	0.287
Stage			
IV vs. III	18.4 vs. 26.6	1.388(0.647,2.976)	0.399
Histology			
Adenocarcinoma vs. Squamous	16.1 vs. 26.0	1.550(0.874,2.749)	0.134
Smoke			
Yes vs. No	22.4 vs. 16.6	0.987(0.566,1.723)	0.964
Brain metastasis			
Yes vs. No	13.3 vs. 24.4	1.961(1.087,3.540)	0.025*
Liver metastasis			
Yes vs. No	13.4 vs. 24.4	2.260(1.194,4.276)	0.012*
Bone metastasis			
Yes vs. No	15.4 vs. 26.0	2.021(1.152,3.543)	0.014*
Pleural effusion			
Yes vs. No	15.0 vs. 22.4	1.816(0.893,3.692)	0.100
Treatment lines			
1line vs. 2 lines vs. ≥ 3 lines^a^	NA vs. 24.4 vs. 14.7	0.446(0.106, 1.877)	0.271
		0.535(0.296, 0.969)	0.039*
PDL1 expression			
Negative vs. Positive	16.6 vs. 22.4	1.157(0.663,2.020)	0.608
Negative vs. Low. vs. High^a^	16.6 vs. 18.2 vs. 26.8	1.357 (0.614,3.001)	0.451
		1.291 (0.543,3.071)	0.563
Gene mutation			
Wild vs. EGFR	22.6 vs. 12.7	0.485(0.249,0.945)	0.033*
Wild vs. EGFR vs. KRAS^a^	18.6 vs. 12.7 vs. 24.4	1.688(0.683, 4.172)	0.257
		3.029(1.145,8.011)	0.026

*NSCLC* Non-small cell lung cancer, *mOS* median overall survival, *HR* Hazard rate, *CI* Confidence interval, *Squamous* Squamous cell carcinoma, *PD-L1* (programmed death ligand 1) expression, Negative, 0%; Positive, ≥ 1%; Low, 1%-49%; High, ≥ 50%. *EGFR* (epidermal growth factor receptor), including classical EGFR mutation and nonclassical EGFR mutation, *KRAS* Kirsten Rat Sarcoma Viral Oncogene Homologue

^*^significance *p* values

^a^the last group served as a control

### Treatment responses

Of the 85 NSCLC patients with ICIs combined antiangiogenic therapy, 28 achieved PR, 43 achieved SD, and 14 achieved PR. The ORR was 32.9%, and the DCR was 83.5% (Table 5). PD-LI expression was associated with treatment responses in advanced NSCLC patients (*p* = 0.000), both in adenocarcinoma (*p* = 0.007) and squamous cell carcinoma (*p* = 0.049). Notably, 1 patient obtained SD and 1 patient obtained PD for each pair of patients with ALK fusion, with BRAF V600E mutation, and with HER-2 exon 20 insertion mutation.

**Table 5:** Relationship between clinical characteristics of NSCLC patients and treatment responses

Characters	Case	CR	PR	SD	PD	χ2	*p* value
NSCLC	85	0	28	43	14		
Gender						4.491	0.106
Female	19	0	4	9	6		
Male	66	0	24	34	8		
Age						2.169	0.338
< 60	40	0	16	17	7		
≥60	45	0	12	26	7		
Brain metastasis						5.444	0.066
No	63	0	21	35	7		
Yes	22	0	7	8	7		
Liver metastasis						3.766	0.152
No	70	0	24	37	9		
Yes	15	0	4	6	5		
Bone metastasis						1.104	0.576
No	52	0	19	24	9		
Yes	33	0	9	19	5		
Pleural effusion						1.125	0.570
No	71	0	25	36	40		
Yes	14	0	3	7	4		
Smoke						2.635	0.268
No	44	0	13	21	10		
Yes	41	0	15	22	4		
Histology						3.806	0.149
Adenocarcinoma	48		16	21	11		
Squamous cell carcinoma	37		12	22	3		
Treatment line						8.504	0.075
1 line	5	0	3	2	0		
2 lines	35	0	13	20	2		
≥ 3 lines	45	0	12	21	12		
PDL1 expression						22.123	0.000*
Negative	44	0	8	29	7		
Low	28	0	9	14	5		
High	13	0	11	0	2		
Gene mutation						10.636	0.100
Wild	36	0	10	20	6		
EGFR	17	0	2	10	5		
KRAS	14	0	8	4	2		
Unknown	18	0	8	9	1		
Adenocarcinoma	48	0	16	21	11		
PDL1 expression						14.032	0.007*
Negative	26	0	5	15	6		
Low	12	0	3	6	3		
High	10	0	8	0	2		
Gene mutation						8.183	0.085
Wild	19	0	6	8	5		
EGFR	16	0	2	9	5		
KRAS	13	0	8	4	1		
Squamous cell carcinoma	37	0	12	22	3		
PD L1 expression						9.538	0.049*
Negative	18	0	3	14	1		
Low	16	0	6	8	2		
High	3	0	3	0	0		

*NSCLC* Non-small cell lung cancer, *CR* Complete response, *PR* Partial response, *SD* Stable disease, *PD* Progressive disease. *EGFR* (epidermal growth factor receptor), including classical EGFR mutation and nonclassical EGFR mutation, *KRAS*, Kirsten Rat Sarcoma Viral Oncogene Homologue. *PD-L1* (programmed death ligand 1) expression, Negative, 0%; Low, 1%-49%; High, ≥ 50%

^*^significance *p* values

### Safety

AEs of different grades occurred in 92.9% (79/85) of the 85 advanced NSCLC patients, most of whom had mild grade 1/2 AEs, and no grade 5 fatal AEs occurred. The most common adverse reactions were fatigue (32.9%), proliferation of capillaries (31.8%), hypotension (29.4%), proteinuria (27.1%), abnormal thyroid function (25.9%), and hand-foot syndrome (23.5%) (Table 6). Grade 3/4 AEs were observed in 32.9% (28/85) of patients, and the most common was hypotension (4.7%), which could be controlled with aggressive medication.

**Table 6:** AEs in advanced NSCLC patients who received combination therapy with PD-1 inhibitors and antiangiogenic agents

Adverse events	Any Grade n (%)	≥ Grade 3(n/%)
Fatigue	28(32.9)	1(1.2)
Proliferation of capillaries	27(31.8)	0(0)
Hypertension	25(29.4)	4(4.7)
Proteinuria	23(27.1)	3(3.5)
Abnormal thyroid function	22(25.9)	2(2.4)
Hand-foot syndrome	20(23.5)	3(3.5)
Rash	18(21.2)	3(3.5)
Anorexia	17(20)	0(0)
Dyslipidaemia	15(17.6)	1(1.2)
Thrombocytopenia	14(16.5)	3(3.5)
Hepatic dysfunction	13(15.3)	2(2.4)
Oedema	12(14.1)	1(1.2)
Anaemia	11(12.9)	1(1.2)
Diarrhoea	11(12.9)	0(0)
Haemorrhage	10(11.8)	0(0)
Nausea	10(11.8)	0(0)
Pruritus	9(10.6)	1(1.2)
Oral mucositis	8(9.4)	0(0)
Myalgia	7(8.2)	0(0)
Leukopenia	6(7.1)	1(1.2)
Pneumonitis	5(5.9)	1(1.2)
Hypophysitis	2(2.4)	0(0)
Myocarditis	2(2.4)	1(1.2)
Encephalitis	1(1.2)	0(0)

*AEs* Treatment-related adverse events, *NSCLC* Non-small cell lung cancer, *PD-1* Programmed death 1

## Discussion

How to improve the efficacy of immunotherapy is a hot topic in clinical research. Preclinical studies have confirmed that ICIs combined with antiangiogenic therapy achieve a 1 + 1 > 2 antitumour effect. An increasing number of clinical studies have begun to explore the application prospects of the chemotherapy-free mode in advanced NSCLC [19].

Domestic and international clinical studies revealed that the mPFS of immunotherapy combined with antiangiogenic therapy in the subsequent treatment of advanced NSCLC was approximately 6 months, and the ORR and DCR were approximately 30% and 80%, respectively [21–24]. Indirectly compared with the previous literature data, the effect of combination therapy is superior to ICI monotherapy, and the mPFS of ICI monotherapy in subsequent therapy in advanced NSCLC was less than 4 months, and the response rate was only approximately 20% [25–27].

In our present study, we retrospectively analysed the efficacy and safety of 85 NSCLC patients who received ICIs combined with antiangiogenic therapy. A total of 94.1% (80/85) of NSCLC patients received second-line and subsequent treatment, the mPFS was 7.5 months, and the ORR and DCR were 31.25% and 82.5%, respectively. The research data of our centre were basically consistent with the real data reported in the past and were slightly better. A 2-centre, retrospective study in the real world revealed that 57 previously treated advanced NSCLC patients who received any PD-1 antibody combined with antiangiogenic drugs exhibited a PFS of 4.2 months and a DCR of 63.2% [28].

A retrospective analysis of 67 advanced NSCLC patients who had previously received PD-1 antibody in combination with anlotinib showed that 19 patients had PR (28.4%), 39 had SD (58.2%) and 9 had PD (13.4%). The mPFS was 6.9 months, and the OS was 14.5 months. The study also found that the benefit of anti-PD-1 plus anlotinib was also observed in patients with EGFR mutation positivity, liver metastases, and brain metastases [29]. In our present study, NSCLC patients with brain metastasis and bone metastasis had shorter PFS and OS, and patients with liver metastasis and EGFR mutations had shorter OS. Although previous studies have found that patients with EGFR mutations do not respond well to immunotherapy [25, 27, 30, 31], in this study, the combination of ICIs and antiangiogenic therapy also achieved a PFS of 4.4 months and an OS of 12.7 months. Among the 17 patients with EGFR mutations, 2 achieved PR, and 10 achieved SD, with a DCR of 70.6%. This study suggested that immunotherapy combined with antiangiogenic therapy can be an option for patients with EGFR mutations after drug resistance, as shown by Impower 150 [15] and ORIENT-31 [16]. KRAS mutations have been linked to better immunotherapy responses in lung cancer [32–34]. Our study showed that patients with KRAS mutations had longer PFS (9.4 months) and OS (24.4 months).

In addition, it was found that patients with high PD-L1 expression were more likely to obtain PR in the combination regimen, a result consistent with the previous conclusion that PD-L1 expression predicts the efficacy of immunotherapy [27, 35]. However, both the PD-L1 high expression group and the positive group had longer PFS and OS. There was no significant difference. Therefore, whether PD-L1 expression status can be used as a predictor of the efficacy of combination treatment modes needs to be further confirmed by large-sample, prospective clinical studies.

Advanced NSCLC patients who received ICIs combined with antiangiogenic therapy at or above 3 lines achieved a PFS of 5.5 months and an OS of 14.7 months. These data could be compared with the results of a retrospective study of 30 samples from Xu et al., in which the mPFS was 5.0 months and the mOS was 14.3 months. Similarly, it was also found that patients with higher PD-L1 expression had longer PFS, but the difference was not statistically significant [36].

Another scholar performed a cohort study of the efficacy and safety of ICIs plus anlotinib versus ICIs alone as the treatment of advanced NSCLC in the real world. The results revealed that the mPFS of patients in the ICI plus anlotinib group was also much longer than that of patients in the ICI monotherapy group (6.37 vs. 3.90 months; *P* < 0.001). Combining ICIs with anlotinib could improve the outcomes of patients with bone metastasis [37]. The above results of this real-world study suggest that ICIs combined with antiangiogenic therapy are a good option for advanced NSCLC patients who have failed first-line therapy.

Surprisingly, the efficacy of immunotherapy combined with antiangiogenic therapy as the first-line treatment for NSCLC patients has also been explored. In the 2019 World Lung Cancer Congress, a study from Professor Han et al. revealed the efficacy of sintilimab combined with anlotinib as the first-line treatment for stage IV NSCLC patients with negative driver genes. A total of 16/22 patients achieved PR, and the ORR was 72.7% (95% CI: 49.8–89.3); DCR was up to 100% (95% C: 84.6–100); mPFS was 15 months (95% CI: 8.3-NR); mOS data were not mature [38]. Based on the current data, sintilimab combined with anlotinib had a great advantage in the first-line treatment of advanced NSCLC.

In our present study, there were 5 NSCLC patients who received ICIs combined with antiangiogenic therapy as the first-line treatment. The mPFS was 8.0 months, and the mOS was not mature, slightly worse than those shown in the study of Han et al. The reason may be that the general status of the included population was relatively poor, and ICIs combined with antiangiogenic therapy were treated as a compromise protocol. In addition, the number of cases included was so small that the strength of the data was limited.

Currently, a phase III clinical study (NCT04964479) in which TQB-2450 (a humanized monoclonal antibody against PD-L1) is combined with anlotinib versus pembrolizumab as a first-line treatment for advanced NSCLC patients with PD-L1 ≥ 1% is ongoing. It is expected that this study will provide good evidence for anlotinib combined with immunotherapy in the first-line treatment of advanced NSCLC. In summary, immunotherapy combined with antiangiogenic therapy has shown good antitumour effects in the first and posterior lines. However, it is difficult to determine which line is better. An indirect comparison with previous literature indicated that first-line single-agent immunotherapy was superior to second-line immunotherapy [27, 35, 39]. The KEYNOTE-001 study revealed that immunotherapy had a longer mOS in untreated patients than in treated patients (22.3 vs. 10.5 months) [40]. In addition, the PFS2 analysis of the KEYNOTE-024 [39] study also showed that the earlier immunotherapy was used, the better the efficacy. However, whether combined immunotherapy must also be performed as early as possible remains to be further explored.

## Conclusion

Immunotherapy combined with antiangiogenic therapy has been increasingly recognized in advanced NSCLC and has been favoured by many clinicians because of its relatively mild adverse effects. In the present study, we retrospectively analysed the efficacy and safety of immunotherapy combined with antiangiogenic therapy in advanced NSCLC in the real world. Regardless of the number of treatment lines, chemotherapy-free combination therapy was an option for advanced NSCLC patients with good safety and tolerability. In particular, patients without brain metastases, bone metastases, liver metastases, and EGFR mutations had longer OS. Immunotherapy combined with antiangiogenic therapy could also be a good choice for patients with KRAS mutations. In addition, NSCLC patients with high PD-L1 expression were more likely to respond to combination therapy and had longer PFS and OS.

Since this study was a retrospective study based on a small sample, sampling differences may affect the results. In addition, ICIs and antiangiogenic drugs were not qualified in this study, and there could be differences in efficacy between different drugs. Finally, this study did not exclude patients who had used ICIs or antiangiogenic drugs alone in the past, and whether cross-line use has an effect on the efficacy of immunotherapy combined with antiangiogenic therapy remains to be further explored.

In the future, more exploration is needed into how to screen the advantaged population. Additionally, more phase III clinical studies are needed to verify the feasibility of clinical application and provide survival benefits to more patients.

## Data Availability

The datasets generated during and/or analysed during the current study are available from the corresponding author on reasonable request.
